# Multilayer all-polymer metasurface stacked on optical fiber via sequential micro-punching process

**DOI:** 10.1515/nanoph-2022-0762

**Published:** 2023-02-08

**Authors:** Moohyuk Kim, Nu-Ri Park, Aran Yu, Jin Tae Kim, Minseok Jeon, Seung-Woo Jeon, Sang-Wook Han, Myung-Ki Kim

**Affiliations:** KU-KIST Graduate School of Converging Science and Technology, Korea University, Seoul, 02841 Republic of Korea; Quantum Technology Research Department, Electronics and Telecommunications Research Institute (ETRI), Daejeon, 34129, Republic of Korea; Center for Quantum Information, Korea Institute of Science and Technology (KIST), Seoul, 02792, Republic of Korea; Division of Nanoscience and Technology, KIST School, Korea University of Science and Technology (UST), Seoul, 02792, Republic of Korea

**Keywords:** metalenses, metasurface-on-fiber, metasurfaces, OAM metasurfaces, polymer metasurfaces

## Abstract

Metasurface technology is revolutionizing the field of optics and pursuing expanded functions via technical developments, such as the integration of multiple metasurfaces with optical fibers. Despite several attempts to realize metasurface-on-fiber platforms, negligible fiber-facet areas pose a serious obstacle to efficient and precise fabrication. Herein, we demonstrate a novel sequential micro-punching process that enables rapid and precise stacking of multiple polymer metasurfaces on the end face of a single-mode optical fiber. Mesh-type nanohole metasurfaces are fabricated on a 1.8-μm-thick polymethyl methacrylate (PMMA) layer via e-beam lithography, and the PMMA layer is separated from the substrate and prepared in the form of a membrane using the external frame. Furthermore, the PMMA metasurfaces are sequentially punched through the fiber and stacked on top. Employing a micro-punching process, we demonstrate highly efficient all-polymer metalenses and orbital angular momentum (OAM) metasurfaces coupled with single-mode fibers operating in the telecommunication band. A 1550 nm laser beam passing through three metalens layers stacked on the fiber is focused at a distance of 135 μm with 83% efficiency. In addition, the 1550 nm beam passing through three OAM metasurfaces on the fiber is converted into a perfect vortex beam with a topological charge of 3. We believe that our proposed micro-punching process will cause a breakthrough in the fabrication of metasurface-integrated optical fibers that will be utilized in a wide range of applications.

## 1 Introduction

Metamaterials, comprising periodic three-dimensional subwavelength metallic/dielectric structures, are artificial materials that can realize unprecedented electromagnetic properties, such as ultra-high refractive index [1], negative refractive index [2], and electromagnetic clocking [3, 4]. However, these attractive features of metamaterials require sophisticated three-dimensional fabrication techniques with complex metal wires [5], which make their assembly difficult and costly. Recently, metasurfaces have been spotlighted as a solution that can address the limitations of metamaterials while retaining their distinct optical properties [6], [7], [8], [9], [10]. Metasurfaces are thin two-dimensional planar optical structures that comprise a series of discrete elements called meta-atoms, which can exert remarkable abilities of electromagnetic wavefront manipulation introduced by the functional arrangement of meta-atomic structures. Advances in metasurface technology over the past decade have revolutionized the fields of optics and photonics, demonstrating several innovative devices such as high-NA metalenses [11], [12], [13], holography [14], invisibility cloaks [15, 16], and broadband absorbers [17, 18].

Metasurfaces are usually fabricated on thin films using standard lithography and nanoimprinting processes; however, recently the fabrication of metasurfaces on optical fiber facets has garnered significant attention [19, 20]. Optical fibers are an excellent platform for combining metasurface features because of their light propagation flexibility, biocompatibility, and physical robustness, which will have a significant impact on various applications, such as environmental sensing and clinical diagnostics [21, 22]. However, despite the fact that the optical fiber provides a planar cross-section suitable for combining with a thin metasurface, there is great difficulty in applying the existing nanotechnology owing to the limited size of the optical fiber facet. Currently, most fiber-based metasurfaces are fabricated directly on the fiber facets using focused ion-beam milling [23], [24], [25] or e-beam lithography followed by etching processes [26], [27], [28], or by patterning nanostructures with laser writing techniques using femtosecond laser pulses or two-photon polymerization [29], [30], [31]. However, these methods have limitations, such as a slow fabrication process, difficulty in fabricating elaborately on small fiber tips (e.g., single-mode fibers), and difficulty in continuously stacking add-on metasurfaces onto prefabricated structures. Recently, it has been proposed that nano-transfer techniques transfer meta structures to small fiber tips [32, 33]. This method employs a process of transferring nanopatterned meta-atoms prefabricated on a detachable thin metal layer to a fiber tip, which has the advantage of continuously transferring various patterns and maintaining stable patterning resolution. However, defects in the pattern intermittently occur during the transfer process and continuous multiple stacking is unstable.

In this study, we propose and demonstrate a novel sequential micro-punching process that can rapidly and precisely transfer multiple polymer metasurfaces onto the end face of a single-mode optical fiber. Mesh-type metasurfaces with nanoholes (diameter = 400 nm–1.1 μm) are fabricated on a 1.8-μm-thick polymethyl methacrylate (PMMA) layer using e-beam lithography (EBL), and the PMMA layer is transferred to an apertured polydimethylsiloxane (PDMS) frame. Furthermore, the PMMA metasurfaces are mechanically punched using the aligned fiber. Via this punching process, multiple PMMA metasurfaces are sequentially stacked on the end face of the fiber. Employing the micro-punching process, we demonstrated highly efficient all-polymer metalenses and orbital angular momentum (OAM) metasurfaces coupled to single-mode fibers operating in the telecommunication band. We observed that a 1550 nm laser beam in a single-mode fiber was focused at a distance of 135 μm with 83% efficiency when passing through three metalens layers stacked on the fiber, and was converted into a perfect vortex beam with a topological charge of 3 when passing through three OAM metasurfaces on the fiber.

## 2 Multilayer all-polymer metasurface on fiber

**Figure 1:**
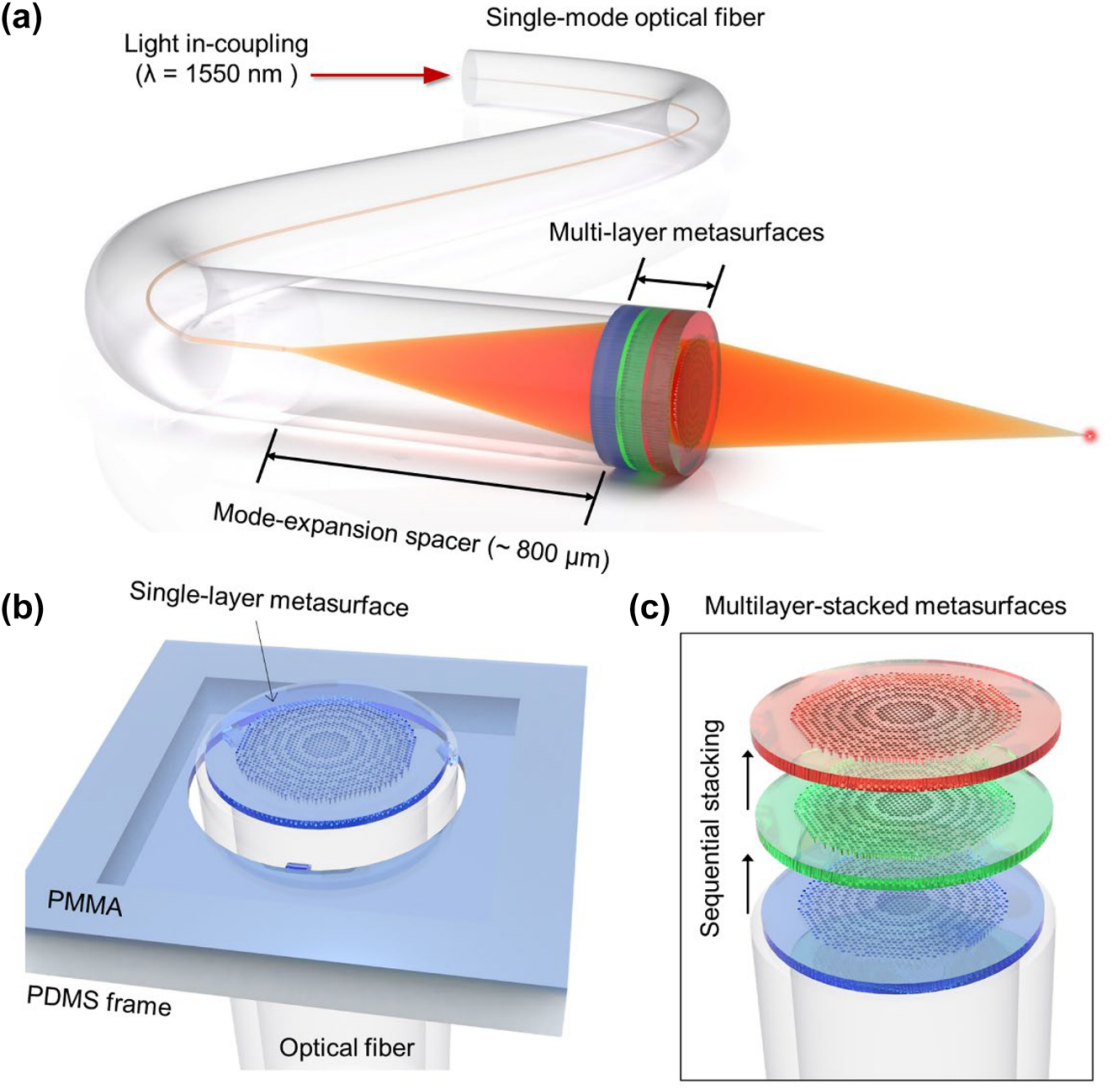
Multilayer all-polymer metasurface stacked on optical fiber. (a) Schematic of the proposed multilayer metasurface-on-fiber platform in which multiple metasurface layers are stacked on the end face of an optical fiber. Here, multimode fiber is connected between the single-mode fiber and metasurface with a length of approximately 800 μm to induce the broadening of the beam. (b) Schematic of the micro-punching process that enables stacking of a PMMA metasurface on the end face of the fiber. (c) Sequential micro-punching process in which multiple free-standing PMMA metasurfaces are stacked onto the end face of the fiber.

The combinatorial use of multiple metasurfaces allows for the expansion of the degree of freedom of metasurface functions, and their efficient integration with optical fibers will allow more freedom in the utilization of expanded functions. Figure 1(a) illustrates a schematic of the proposed multilayer metasurface-on-fiber platform in which multiple metasurface layers are stacked on the end face of an optical fiber. In this platform, the beam propagating in a low-loss single-mode fiber (SMF) with a core diameter of 9 μm is emitted as a manipulated wavefront by passing through multiple metasurfaces stacked at the end of the fiber. The mode field diameter (MFD) of the beam in the SMF at a wavelength (*λ*) of 1550 nm is approximately 10 μm, while the beam diameter that needs to cover the metasurface to achieve the full performance of the metasurface is at least 100 μm; therefore, the beam should expand approximately ten times or more from the end of the SMF to the metasurface. To this end, a multimode fiber (MMF, 0.22 NA) with a core diameter of 105 μm is connected between the SMF and metasurface with a length of approximately 800 μm to induce the broadening of the beam. Here, SMF and MMF have the same cladding size of 125 μm and are neatly joined by fusion splicing; hence, a seamless connection between the two fibers is achieved [Supplementary Note 1]. In addition, if the core diameter of the MMF is large enough, the Gaussian beam shape is maintained during beam expansion [27].

Fabricating multilayer metasurfaces on an optical fiber facet using conventional nanofabrication techniques encounters complex fabrication steps, such as: (1) depositing a metasurface layer with a certain thickness on the end face of the fiber, (2) coating e-beam resist on the fiber facet, (3) EBL with a special holder to level the fiber facet, (4) dry etching to the fiber facet, and (5) repeating the previous works to stack multilayer metasurfaces on the fiber [34, 35]. These processes are inferior in processability because of the limited area of the fiber facet, and in addition, they require extensive time and high costs owing to their complex and several process steps. The introduction of polymer-based metasurfaces can simplify the fabrication steps. In particular, if e-beam resists such as PMMA are directly used as a metasurface material, the process steps can be drastically reduced because nanostructures are produced via a simple development process after EBL. PMMA has high transmittance for visible and near-infrared beams, excellent flexibility, high acid resistance and chemical stability, high mechanical strength [36], and can be processed in the form of a membrane.

We propose a PMMA-based micro-punching process that allows the continuous stacking of various PMMA metasurfaces onto the fiber. As illustrated in Figure 1(b), the PMMA layer containing various metasurfaces is placed on the PDMS frame and mechanically punched through the fiber in succession; therefore, each PMMA metasurface is stacked directly to the end face of the fiber. Nanostructures of the PMMA metasurfaces are fabricated through EBL, and multiple metasurfaces are successively punched through sequential micro-punching; therefore, the whole process is fast and cost-effective. For the micro-punching process, the metasurface must have a free-standing mesh-like structure without a substrate. In addition, the edges of the metasurfaces should be pierced in a ring form to separate them from the PMMA layer and transfer them to the fiber, and a few thin tethers are needed to support them weakly on the layer. Under these conditions, multilayer metasurfaces can be stably and quickly stacked onto the end face of the fiber by sequentially punching multiple free-standing PMMA metasurfaces, as illustrated in Figure 1(c).

## 3 Micro-punching process

**Figure 2:**
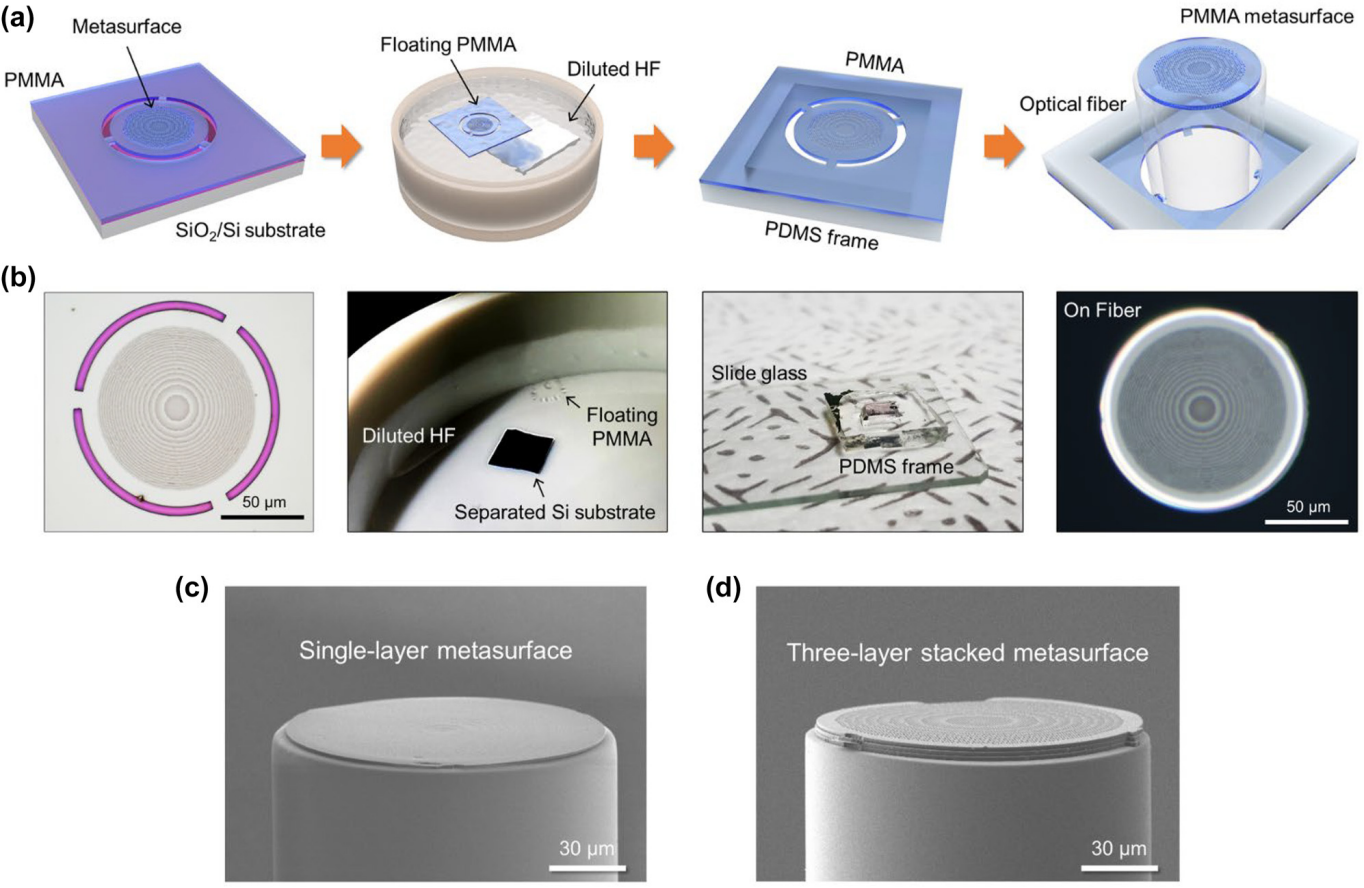
Fabrication of multilayer PMMA metasurface on fiber via the micro-punching process. (a) Four major fabrication steps: (i) fabrication of the PMMA metasurfaces via the EBL process, (ii) separation of the PMMA layer from the substrate, (iii) preparation of the PMMA membrane using the PDMS frame, and (iv) punching of the PMMA metasurfaces via the aligned fiber. (b) Images and photographs corresponding to the process step in (a). (c) Scanning electron microscopy (SEM) image of the fabricated sample in which a single-layer PMMA metasurface is stacked onto the fiber. (d) SEM image of the fabricated sample in which three-layer PMMA metasurfaces are stacked onto the fiber.

As illustrated in Figure 2(a) and (b), we passed through four major steps for the micro-punching process: (1) fabrication of the PMMA metasurfaces via the EBL process, (2) separation of the PMMA layer from the substrate, (3) preparation of the PMMA membrane using the PDMS frame, and (4) punching of the PMMA metasurfaces through the aligned fiber. First, a 1.8-μm-thick PMMA is coated on the Si substrate with a thermally grown 285-nm-thick SiO_2_ layer on top. Here, a 1.8 μm thickness of PMMA is close to the maximum thickness that can be nano-pattered through EBL [37]. Furthermore, various metasurfaces are nano-patterned through EBL and structured through a developing process. At the edges of the metasurfaces, 5-μm-wide ring patterns with a 120 μm diameter slightly smaller than a fiber cladding diameter of 125 μm are introduced, and several 10-μm-wide tethers are left inside the ring. Furthermore, the patterned PMMA layer is separated from the substrate by removing the SiO_2_ sacrificial layer of the SiO_2_/Si substrate using a diluted HF (HF:H_2_O = 1:4, DHF) solution, and the PMMA film is floated on the DHF solution. The PMMA film floating in the DHF solution is rinsed by immersing it in deionized (DI) water, taken out using an Au boat, and then transferred to a PDMS frame having an open aperture to prepare for punching. After making sure that the open area of the PDMS frame and PMMA film are aligned correctly, the PDMS frame is pressed to produce sufficient adhesion between the PMMA and PDMS. Here, a certain amount of DI water film exists between the PMMA and Au boat, which allows for easy separation of PMMA and Au, so that the PMMA film can be readily transferred to the PDMS frame [Supplementary Note 2, Supplementary Video 1]. Because the PMMA is flexible and resistant to external stress, nanostructures fabricated on the PMMA are safely transferred to the PDMS frame without damage. Furthermore, a visible laser (*λ* = 635 nm) is applied through one port of the optical fiber. Using the beam emitted from the cleaved fiber on the opposite side, the fiber axis and center of the metasurface are precisely aligned with each other. Here, MMF with a core diameter of 105 μm is connected to the SMF and cut into 800 μm lengths. Afterward, the fiber is aligned with the edge ring of the PMMA metasurface and punches the metasurface to stack it onto the end face of the fiber. The PMMA metasurface is elastically stacked on the fiber with the flexibility of the material even if there are slight irregularities at the end of the fiber. In this process, van der Waals force and surface tension are simultaneously applied to stably attach the metasurface to the fiber surface. Because the PMMA metasurface is relatively large (diameter ∼ 120 μm), the adhesion by van der Waals force solely is not strong enough to stably adhere the metasurface to the fiber facet, which is also sensitive to environmental conditions such as ambient temperature and humidity. To compensate for this, the surface tension that attracts the surfaces as the liquid dries is additionally applied so that the adhesion stability can be further increased [Supplementary Video 2]. Figure 2(c) illustrates a scanning electron microscopy (SEM) image of the fabricated sample in which a single 1.8-μm-thick PMMA metasurface is stably stacked on the end face of the optical fiber. In addition, by repeatedly punching various PMMA metasurfaces present in the PMMA film using the same fiber, multiple PMMA metasurfaces can be stably multilayered on the fiber. Finally, the stacked PMMA metasurfaces were irradiated with UV beams of an intensity of 1.4 W/cm^2^ for 10 min to enhance the durability of the PMMA. Figure 2(d) illustrates the fabricated sample in which three PMMA metasurfaces are well aligned and stably stacked on the end face of the optical fiber.

## 4 Telecom-band multilayer all-polymer metasurface on fiber

A potential application of the multilayer metasurface-on-fiber is the realization of high-efficiency polymer-based metasurfaces operating in telecommunication bands and their efficient coupling with SMFs. Recently, several attempts to implement metasurfaces operating at telecommunication wavelengths (e.g., *λ* = 1550 nm) have been reported, most of which have been demonstrated using silicon-based metasurfaces with high refractive index [38], [39], [40]. Because the refractive index of silicon (*n* = 3.48) is approximately 2.3 times larger than that of polymer (*n* ∼ 1.5), a full 2π phase shift of the beam can be achieved at the telecommunication wavelength even with a thin thickness of less than 1 μm, and this thickness is within the range in which nanostructures of metasurfaces can be manufactured with conventional nanofabrication techniques. Accordingly, silicon has been widely utilized as a metasurface material for communication bands; however, silicon has strong reflection loss at the interface owing to the high refractive index difference with the outside, and it is difficult to fabricate metastructures connected to optical fibers. There has been an increased demand to implement polymer-based metasurfaces that operate in communication bands with high transmittance and excellent flexibility; however, there is a critical obstacle, in that the polymer must be several micrometers thick to cover the complete 2π phase shift. Such a thickness has limitations in fabricating nanostructures with conventional nanofabrication techniques. Recently, a study on implementing a 3D polymer metastructure on the surface of a fiber using 3D laser writing techniques based on two-photon polymerization has been reported [30, 31]. However, the fabrication resolution is not as accurate as 1 μm, and the process is long and costly. In contrast, our proposed micro-punching process allows multilayer PMMA metasurfaces fabricated by EBL to be stacked onto the fiber facet with the desired total thickness, enabling a fast and accurate realization of a polymer-based metasurface-on-fiber that operates in communication bands.

**Figure 3:**
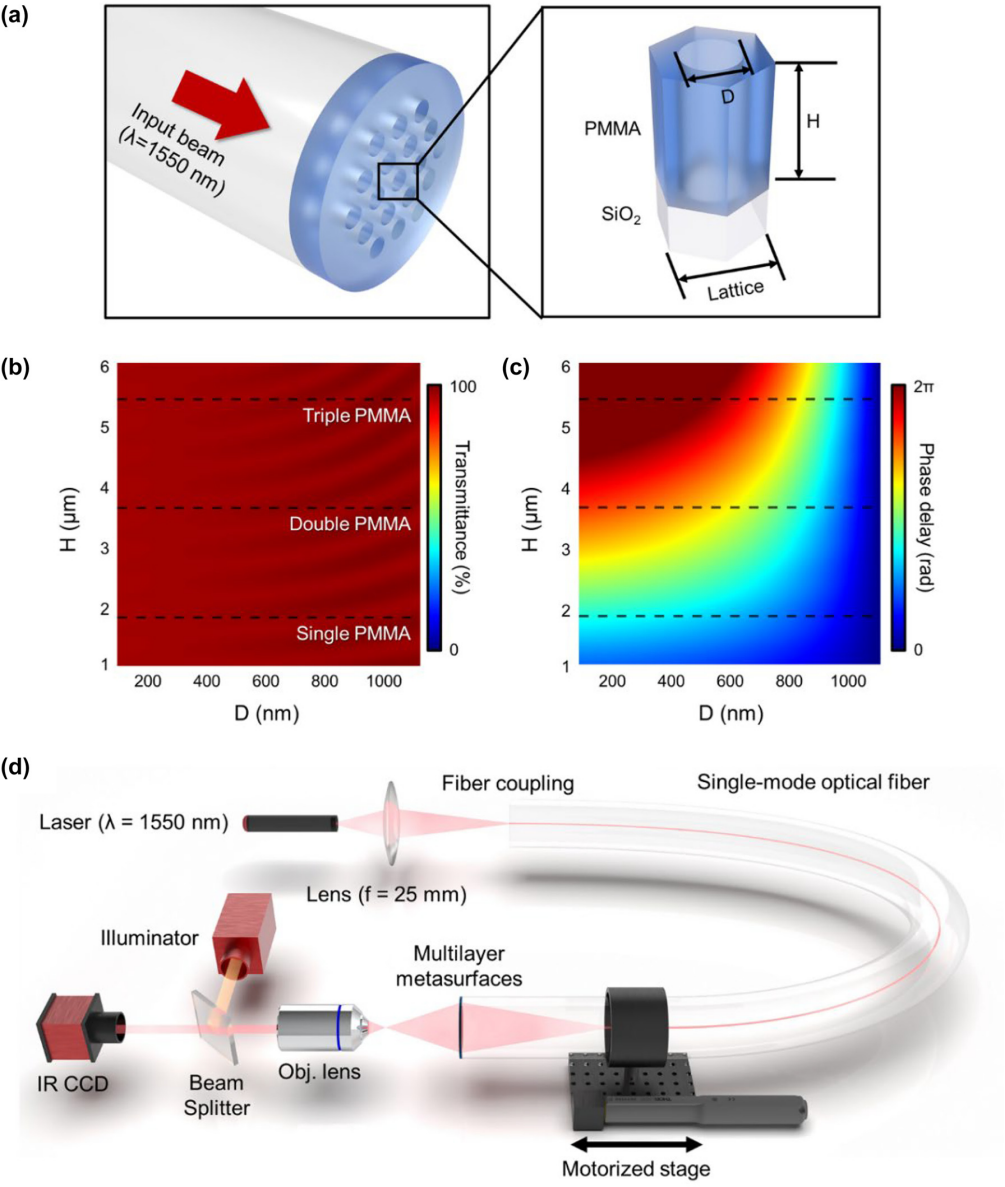
Simulation of PMMA meta-atoms with nanoholes and experimental setup (a) PMMA meta-atom with nanohole. The meta-atom is designed as a hexagonal unit cell, and the unit-cell size (lattice) is fixed at 1200 nm. (b, c) Calculated transmittance and phase delay for a 1550 nm beam as the PMMA thickness (*H* = 1.0–6.0 μm) and hole diameter (*D* = 100–1100 nm) are varied, respectively. (d) Experimental setup to characterize the performance of the telecom-band PMMA metasurface-on-fiber.

Figure 3(a) illustrates the PMMA metasurface with nanoholes to which the micro-punching process is applicable. Because the PMMA nanostructures should be free-standing even after the PMMA layer is separated from the substrate, we designed a mesh-like metasurface with nanoholes in PMMA. The meta-atom was designed in a hexagonal unit cell to increase the number density of meta-atoms per unit area. And the unit-cell size (lattice) was selected to be 1200 nm, which is within a range where the high transmittance does not significantly vary with the size of the nanoholes [Supplementary Note 3]. Figure 3(b) and (c) illustrate the calculated transmittance and phase delay for a 1550 nm beam incident from an optical fiber as the PMMA thickness (*H* = 1.0–6.0 μm) and hole diameter (*D* = 100–1100 nm) are varied, respectively. Here, the calculations were performed by finite-difference time-domain (FDTD) simulations. As illustrated in Figure 3(b), the transmittance over the entire calculation range of *H* and *D* exhibits as high as 96% or more, because the refractive index of PMMA (*n* = 1.4971) is similar to that of SiO_2_. Here, the larger the air hole in the meta-atom, the higher the transmittance, reaching approximately 99%. However, as illustrated in Figure 3(c), the phase delay does not always cover a full 2π shift by *D* for all *H*. For a single-layer PMMA thickness (*H* = 1.8 μm), a 2π phase shift cannot be obtained via *D*, and *H* must be thicker than 4.5 μm to get an entire phase shift. This means that at least three more PMMA metasurfaces (*H* ≥ 1.8 μm × 3) must be stacked to fully work for the 1550 nm beam. Accordingly, we stacked three 1.8-μm-thick PMMA metasurfaces on the end face of the optical fiber using the micro-punching process and demonstrated polymer metalenses and OAM metasurfaces that operate in telecommunication bands.

Figure 3(d) illustrates the experimental setup to characterize the performance of the fabricated telecom-band PMMA metasurface-on-fiber. A 1550 nm laser was incident on one end of a single-mode optical fiber, and the other fiber side with metasurfaces was mounted on a stepper motor to move it at a constant speed along the fiber axis. Furthermore, we measured infrared charge-coupled device (IRCCD) images of the emission beam passing through multilayer metasurfaces as a function of the distance between the metasurface and the image plane of the objective lens. Here, we utilized an objective lens with an NA of 0.42 and an IRCCD with a 320 × 256 pixel array.

## 5 Telecom-band all-polymer metalens on fiber

The most studied application of metasurface-on-fiber is the metalens-on-fiber, which causes a beam emitted through the fiber to be focused at a specific distance. We implemented a high-efficiency polymer metalens-on-fiber that operates in the telecom band by stacking three PMMA metalens layers on the end face of an optical fiber using a sequential micro-punching process. To design a metasurface that serves as a lens that converts an incident plane wave into a focusable spherical wave, we adopted the phase delay map of *φ*_lens_ expressed by Eq. (1) [11].

(1) $$\varphi {\left(x,y\right)}_{\text{lens}}=-\frac{2\pi }{\lambda }\left(\sqrt{x^{2}+y^{2}+f^{2}}-f\right)$$

where (*x*, *y*) denotes the spatial position, *f* is the target focal length of the lens, and *λ* is the operating wavelength of 1550 nm. We set *f* to 100 μm so that the Gaussian beam emitted from the SMF (NA = 0.12) is focused at a distance of 131 μm [Supplementary Note 4]. Based on the calculated *φ*_lens_, we designed metalens by introducing nanoholes with diameters (*D*) corresponding to *φ*_lens_ via Figure 3(c) [Supplementary Note 5]. Here, the diameters of the smallest and largest holes were 400 nm and 1.1 μm, respectively. In addition, we verified via full 3D FDTD simulation that the designed metasurface works as a metalens with 95% efficiency, and when the alignment error of each layer is within approximately 1 μm, the efficiency compared to the perfectly aligned structure was calculated to be over 85% [Supplementary Note 6].

**Figure 4:**
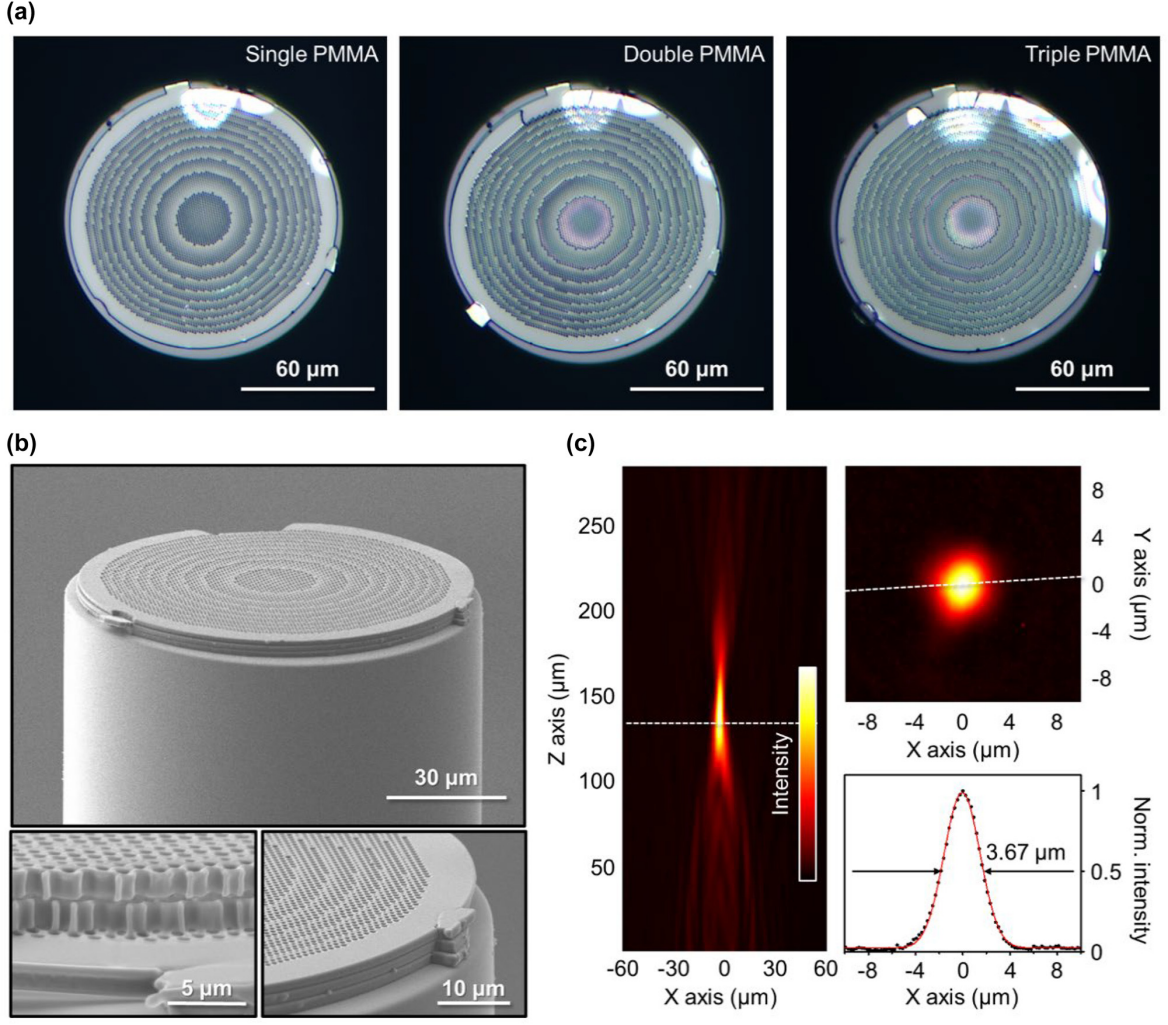
Telecom-band all-polymer metalens on fiber. (a) Microscope images as three identical 1.8-μm-thick PMMA metalenses are being successively stacked one by one onto the fiber. (b) SEM images of fabricated telecom-band PMMA metalens-on-fiber. (c) Characterization of fabricated PMMA metalens-on-fiber. A 1550 nm beam passing through the multilayer PMMA metalens is focused at a distance of approximately 135 μm, which closely matches the designed 130 μm. The transmittance is 83%, and the full width at half maximum (FWHM) of the focused beam is 3.67 μm.

Based on the design, we fabricated a metalens-on-fiber by elaborately stacking three PMMA metasurface layers (total thickness = 5.4 μm) onto the end face of an optical fiber using EBL and sequential micro-punching process. Figure 4(a) illustrates microscope images when three identical 1.8-μm-thick PMMA metalenses were stacked in succession onto the fiber. Here, to precisely align the three metalenses, we stacked them while aligning the positions of the tethers and outermost edges of the layers via an optical microscope. Furthermore, we actively used the surface tension of the remaining water film of each layer for stable attaching between layers [Supplementary Video 2]. The diameter of metalens and outermost diameter of PMMA were measured as 105 μm and 120 μm, respectively. As illustrated in Figure 4(b), the average alignment error of the layered metasurface is measured to be less than 500 nm, which is limited by the resolution of the microscope utilized during the fiber alignment and punching processes.

To characterize the performance of the fabricated metalens-on-fiber structure, we measured IRCCD images of the beam emitted from the fiber as a function of distance using the experimental setup of Figure 3(d) [Supplementary Video 3]. As illustrated in Figure 4(c), a 1550 nm beam passing through the multilayer PMMA metalens was focused to a distance of approximately 135 μm, which closely matches the designed 130 μm. The error in the two distances is estimated to be due to fabrication and misalignment errors that occur during the manufacturing process. The transmittance was measured to be 83%, and the full width at half maximum (FWHM) of the focused beam is 3.67 μm. In addition, we experimentally observed that the single-layer PMMA-stacked metalens works at the visible wavelength of 635 nm [Supplementary Note 7].

## 6 Telecom-band all-polymer OAM metasurface on fiber

Because the metasurface allows manipulation of the spatial wavefront with a high degree of freedom via the two-dimensional arrangement of various meta-atoms, it enables various wavefront modulations for special purposes. Recently, the generation of vortex beams by exciting the OAM modes using metasurfaces has been reported [41]. The generation of OAM modes is particularly interesting in the telecommunications field because OAM modes can be utilized as data channels to expand transmission data capacity. The conventional method of forming a Laguerre–Gaussian (LG) vortex beam using a spiral phase plate (SPP) has a limitation because the beam intensity varies depending on the topological charge (TC), which greatly limits the formation of a perfect vortex (PV) beam [42]. As a solution to this challenge, a method of obtaining a PV beam based on a Bessel–Gaussian (BG) vortex beam using the combination of a spatial light mirror (SLM), an axicon, an interferometer, and a digital micromirror device (DMD) has been proposed [42], [43], [44], [45], [46], [47]; however, their miniaturization and integration remain a challenge. Accordingly, optical fibers with integrated metasurfaces are expected to provide the most promising solution for forming PV OAM beams with a compact platform. We implemented a telecom-band PV beam based on a metasurface-on-fiber platform in which three PMMA OAM metasurfaces were stacked on the fiber facet via the micro-punching process.

In most cases, to form the PV beam, an LG beam is generated via the SPP and converted into the BG beam using the axicon. Furthermore, the BG beam is Fourier transformed using the lens to form the PV beam. The metasurface can integrate these three elements – SPP, axicon, and lens – into one phase plate, thus allowing for perfect miniaturization and integration. The integrated phase profile (*φ*_PV_) generating the PV beam is expressed as the sum of the phase profiles (*φ*_SPP_, *φ*_axicon_, *φ*_lens_) of three elements, and is described by the following equations [48]:

(2) $$\varphi {\left(x,y\right)}_{\text{SPP}}=l\cdot \tan^{-1}\left(\frac{y}{x}\right)$$

(3) $$\varphi {\left(x,y\right)}_{\text{axicon}}=-2\pi \frac{\sqrt{x^{2}+y^{2}}}{d}$$

(4) $$\varphi {\left(x,y\right)}_{\text{PV}}=\varphi {\left(x,y\right)}_{\text{SPP}}+\varphi {\left(x,y\right)}_{\text{axicon}}+\varphi {\left(x,y\right)}_{\text{lens}},$$

where *l* is the TC value and *d* is the periodic length of the axicon design. We set *l* and *d* to 3 and 8 μm, respectively.

**Figure 5:**
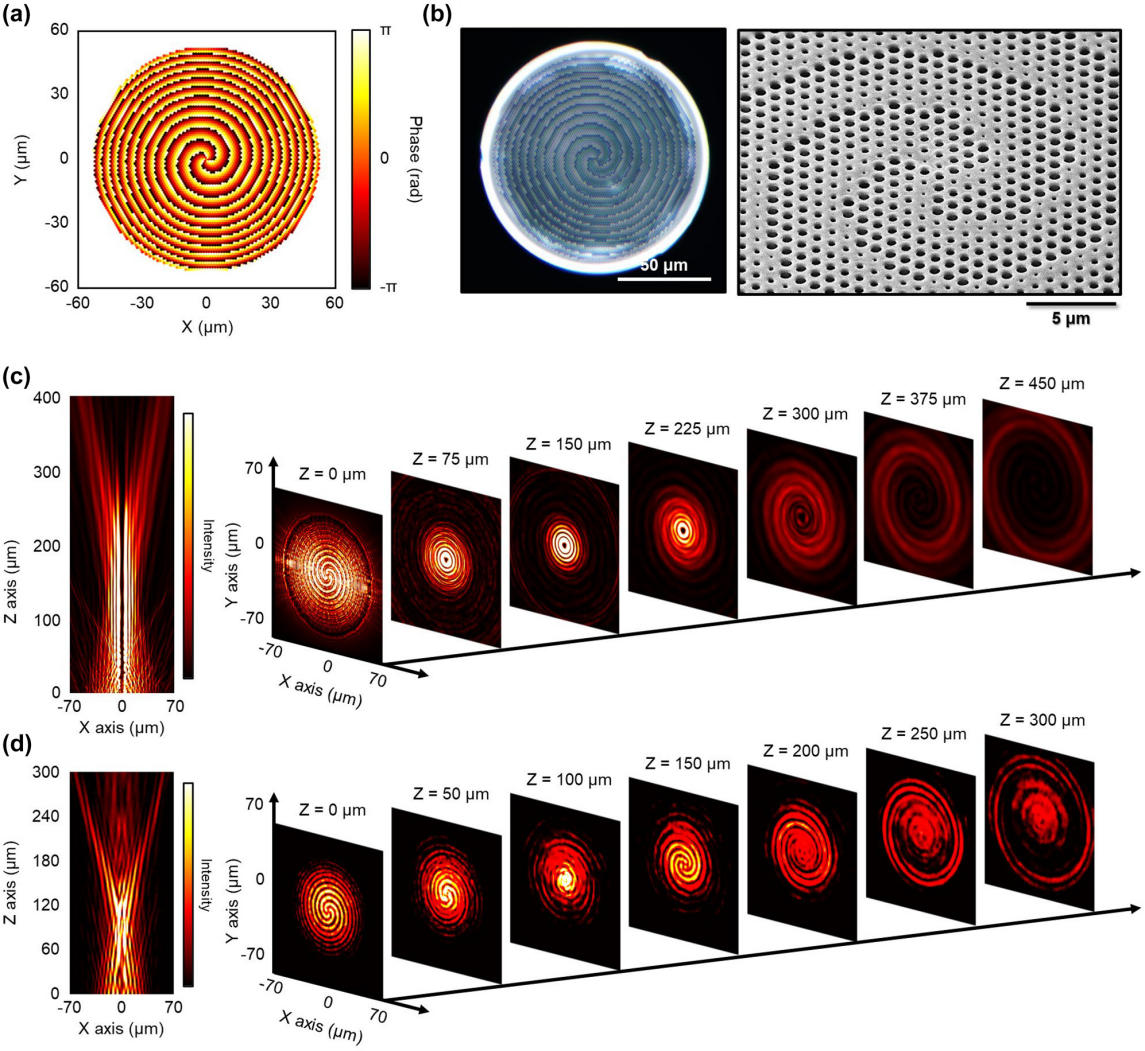
Telecom-band all-polymer OAM metasurface on fiber. (a) Phase map for perfect vortex OAM beam with a topological charge of 3 at a wavelength of 1550 nm. (b) Microscope (left) and SEM (right) images of fabricated telecom-band OAM metasurface-on-fiber. (c) Calculated field intensity profile of a 1550 nm beam passing through the OAM metasurface as a function of distance. (d) Measured IRCCD images of a 1550 nm beam passing through the fabricated OAM metasurface-on-fiber as a function of distance.

Figure 5(a) illustrates the phase profile *φ*_PV_ of the PV OAM with a TC value of 3 calculated by Eqs. (1)–(4). Based on this phase map, we designed and fabricated PMMA OAM metasurfaces by introducing nanoholes with diameters corresponding to *φ*_PV_ into the PMMA layer, and stacked three metasurfaces (total thickness = 5.4 μm) at the end of the fiber via the micro-punching process, as illustrated in Figure 5(b). Furthermore, we confirmed that the OAM metasurface was fabricated with an error of less than 5% via SEM images. Figure 5(c) illustrates the calculated intensity profile of a beam passing through the OAM metalens as a function of distance. It is evident from this figure that the TC ring of the PV beam with a TC value of 3 is being formed by passing through the OAM metasurface. Figure 5(d) illustrates the measured IRCCD images according to the distance of the beam passing through the fabricated OAM metasurface-on-fiber of Figure 5(b). We can observe in this figure that the TC ring of the PV beam with a TC value of 3 is clearly generated, very similar to the simulation result in Figure 5(c) [Supplementary Video 3]. However, unexpected non-zero intensity was observed at the center of the output beam, which may be attributed to fabrication imperfections. The transmittance was measured as 83%, the same as that of the metalens. Via this experiment, we verified that the polymer-based OAM metasurface can be implemented in communication bands and efficiently integrated with the SMF via the micro-punching process. In addition, we observed the formation of a similar PV beam for a 635 nm visible beam in the single-layer PMMA OAM metasurface-on-fiber [Supplementary Note 8]. In this case, a clear observation of zero intensity at the center of the output beam was made.

## 7 Conclusions

We demonstrated a novel sequential micro-punching process that can rapidly and precisely stack multiple PMMA metasurfaces onto the end face of a single-mode optical fiber. This process not only facilitates the fabrication of transparent and flexible polymer metasurfaces with nanometer resolution stacked on optical fibers but also enables the implementation of three-dimensional metastructures with desired thicknesses. We demonstrated highly efficient all-polymer metalenses and OAM metasurfaces coupled with single-mode fibers working for a 1550 nm beam, i.e., a telecom-band beam, via the micro-punching process. Optical fibers combined with metasurface functions are an excellent platform to enhance their functionality and usability in beam engineering. We believe that the proposed micro-punching process will cause a breakthrough in the fabrication of metasurface-integrated optical fibers, which will play a revolutionary role in a wide range of applications including communication, sensing, imaging, and biomedical applications.

## Supplementary Material

Supplementary Material Details

## Supplementary Material

The article contains supplementary material (https://doi.org/10.1515/nanoph-2022-0762).
