# Supplementary material for: Multilayer all-polymer metasurface stacked on optical fiber via sequential micro-punching process
Source: Nanophotonics. 2023 Feb 8;12(13):2359–69. doi: 10.1515/nanoph-2022-0762 (PMC11501433; doi:10.1515/nanoph-2022-0762)
Supplement: Supplementary file 1 — Supplementary Material Details [file j_nanoph-2022-0762_suppl.pdf]

Supplementary Material

Multilayer all-polymer metasurface stacked on optical fiber via sequential micro-punching process

Moohyuk Kim[1], Nu-Ri Park[1], Aran Yu[1], Jin Tae Kim[2], Minseok Jeon[1,3], Seung-Woo Jeon[3], Sang-Wook Han[3,4], and Myung-Ki Kim[1,3]*

[1]*KU-KIST Graduate School of Converging Science and Technology, Korea University, Seoul, 02841 Republic of Korea*
[2]*Quantum Technology Research Department, Electronics and Telecommunications Research Institute (ETRI), Daejeon, 34129, Republic of Korea*
[3]*Center for Quantum Information, Korea Institute of Science and Technology (KIST), Seoul, 02792, Republic of Korea*
[4]*Division of Nanoscience and Technology, KIST School, Korea University of Science and Technology (UST), Seoul, 02792, Republic of Korea*
**rokmk@korea.ac.kr*

Supplementary Note 1: MFD expansion via SMF/MMF connection

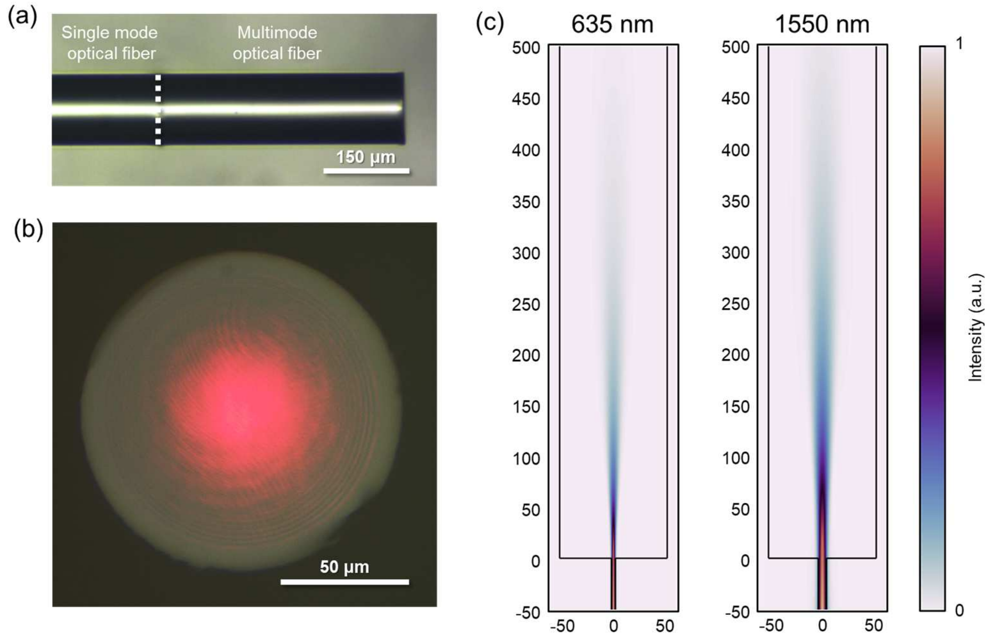

Fig. S1. MFD expansion via SMF/MMF connection: (a) Optical microscope image of a 500-μm long multimode fiber (MMF) spliced to the end of a 635-nm single mode fiber (SMF) (b) Microscope image of expanded mode field diameter (MFD) after a 635-nm wavelength laser beam in SMF (core diameter = 4 μm) passes through a 500-μm length MMF (core diameter = 105 μm) spacer. (c) FDTD simulation results of 635-nm and 1550-nm beams expanding through MMF spacers. Here, the black lines represent the core boundaries of the fibers.

Supplementary Note 2: Fabrication process of PMMA metasurface array with free-standing membrane structure

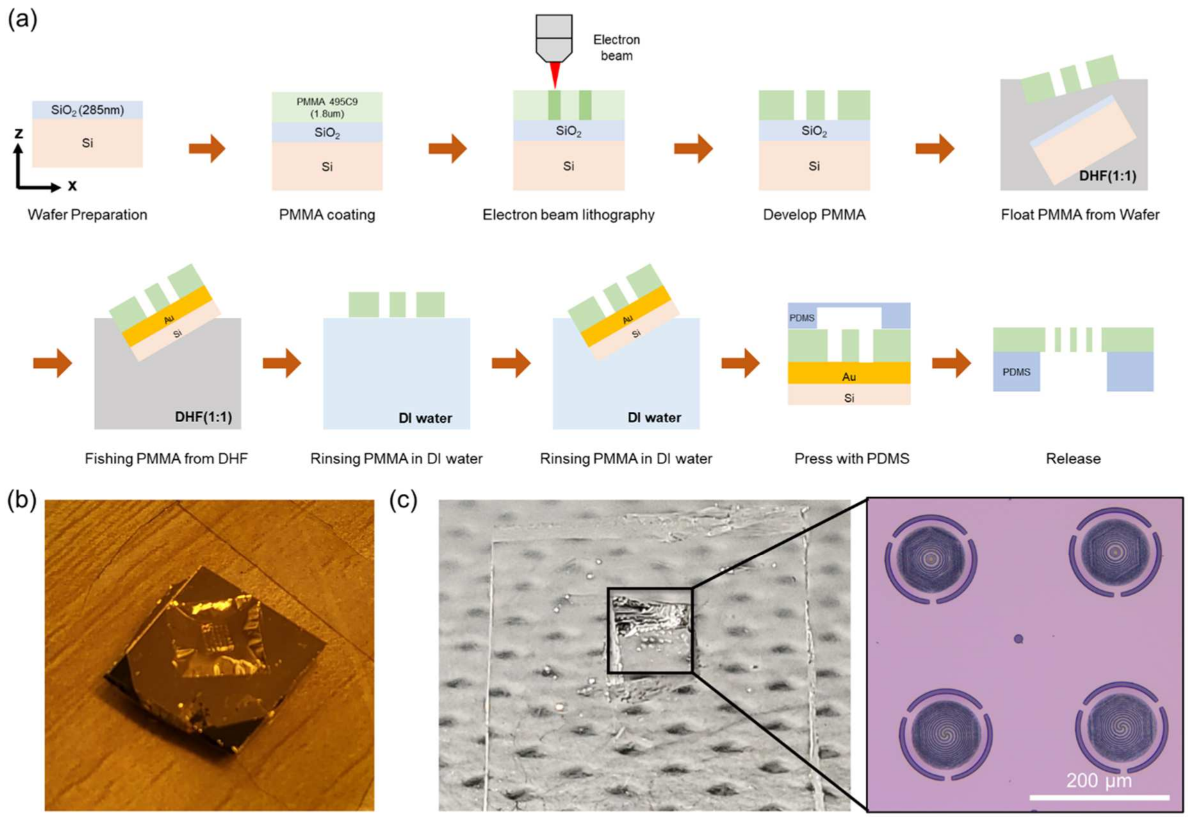

Fig. S2. Fabrication Process of PMMA metasurface array with free-standing membrane structure: (a) Overall schematic of the fabrication process of PMMA metasurfaces array. (b) Image of Patterned PMMA film which is scooped up by Au boat after separation from substrate. (c) Image of transferred PMMA metasurface on perforated PDMS frame. The right image is an optical microscope image of a metasurface array in membrane structure.

Supplementary Note 3: Influence of unit-cell size (lattice) of PMMA meta-atom on output beam characteristics

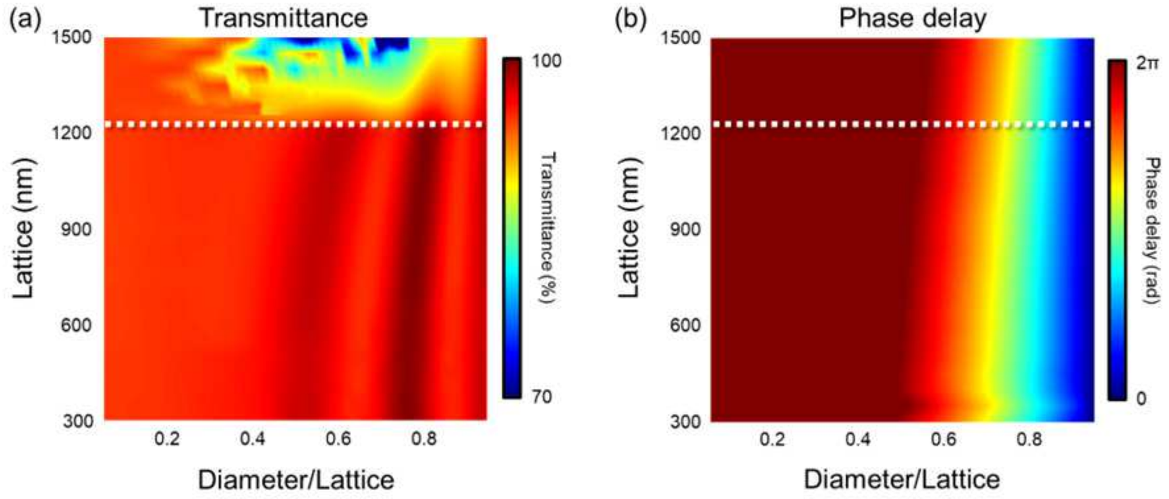

Fig. S3. Influence of unit-cell size (lattice) of PMMA meta-atom on output beam characteristics: Calculated (a) transmittance and (b) phase delay for a 1550-nm beam as the lattice (300-1500 nm) and the diameter-to-lattice ratio (0.05~0.95) of the PMMA metal-atom are varied. Here, the height of the PMMA meta-atom is fixed at 5.4 μm.

Supplementary Note 4: Focal position for an angled incident beam

When an incident beam with an angle θ passes through a lens of focal length f, the focusing position (L) is determined by Eq. (S1). When using parameters f = 100 μm, NA = 0.12, and h = 50 μm, L is calculated to be 131 μm.

$$\frac{1}{L} = \frac{1}{f} - \frac{n_{core} \times \sin\theta}{h} = \frac{1}{f} - \frac{NA}{h} \tag{S1}$$

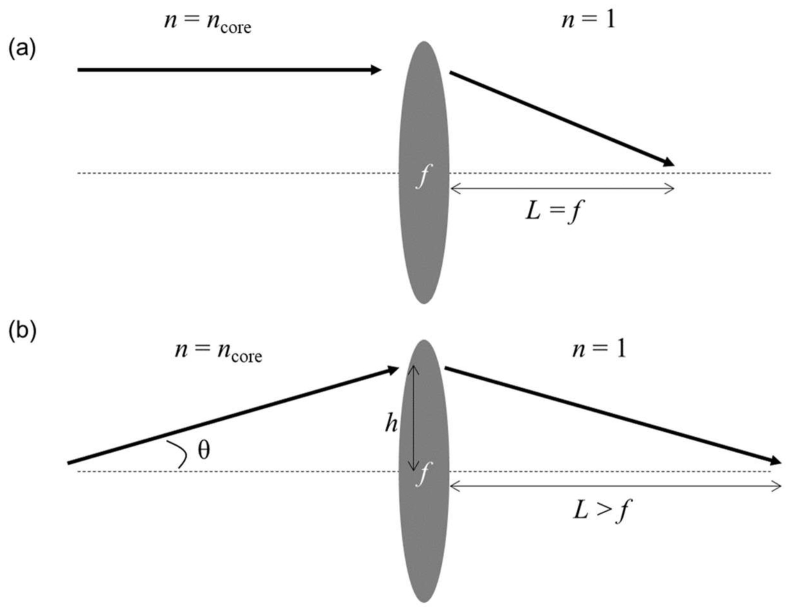

Fig. S4. Focal position for an angled incident beam: The difference in focusing position between (a) when the incident beam is parallel and (b) when the incident beam has an angle θ, for a lens with a focal length *f*.

Supplementary Note 5: Simulation results of PMMA metalens for 1550-nm planewave incident

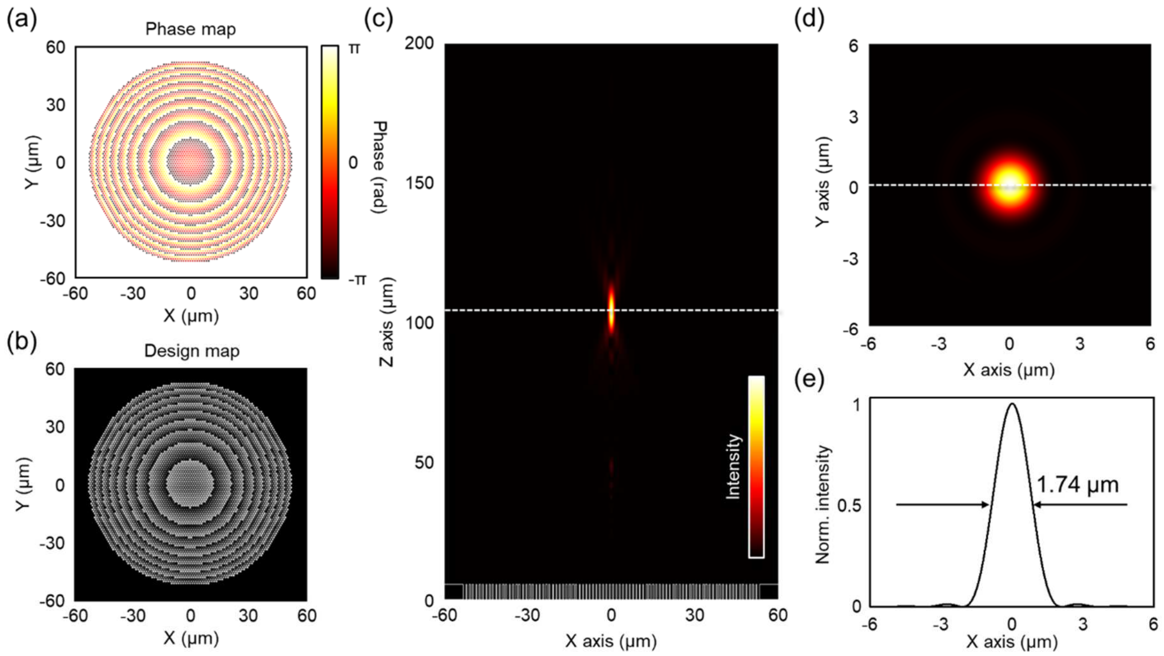

Fig. S5. Simulation results of PMMA metalens for 1550-nm planewave incident: (a) Phase map of PMMA metalens which has a target focal length of 100 μm in 1550 nm wavelength. The metasurface height is 5400 nm and the lattice of each unit cell is 1200 nm. (b) Design map of metalens which is interpolated with figure S3(a) data. The following design map is converted to an electron beam lithography pattern file and then patterned using EBL equipment with designed tethers. (c-e) Simulation result of designed metalens when 1550 nm plane wave light is applied. The near field of metalens up to the height of 100 nm of the surface of metalens is calculated by a 3D FDTD solver, and the far field of metalens is calculated by the vectorial Rayleigh-Sommerfeld integration method. (c) The xz axis graph of the transmitted wavefront. It is observed that the light is focused at 100.8 μm from the surface of the metalens. (d–e) The xy axis graph of the focus plane. The calculated FWHM is 1.74 μm and the calculated transmittance is over 95 %.

Supplementary Note 6: Influence of misalignment in sequential stacking process

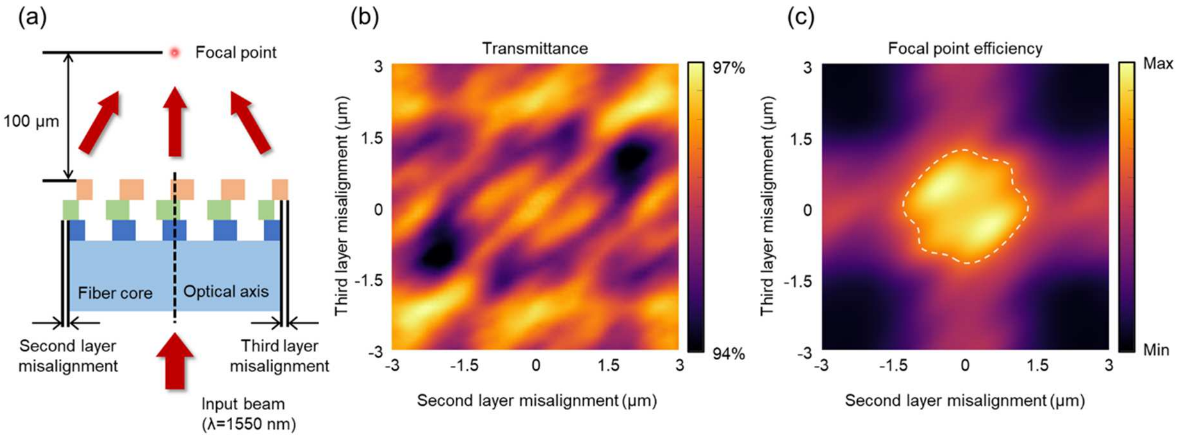

Fig. S6. Influence of misalignment in sequential stacking process: (a) The simulation schematic of misalignment of each layer. The following simulation is calculated by a 2D FDTD solver. We calculated the transmittance and electric field intensity of the focal point difference. (b) The difference in transmittance when misalignment occurred is approximately -3 – 3 μm. (c) The focal point efficiency graph calculated using the electric field intensity difference of the ideal focal point. The ideal focal point is the center of the focal point when there is no alignment error. When the alignment error of each layer is within approximately 1 μm, the efficiency compared to the perfectly aligned structure is calculated to be over 85%.

Supplementary Note 7: Single-layer metalens on fiber in 635 nm wavelength

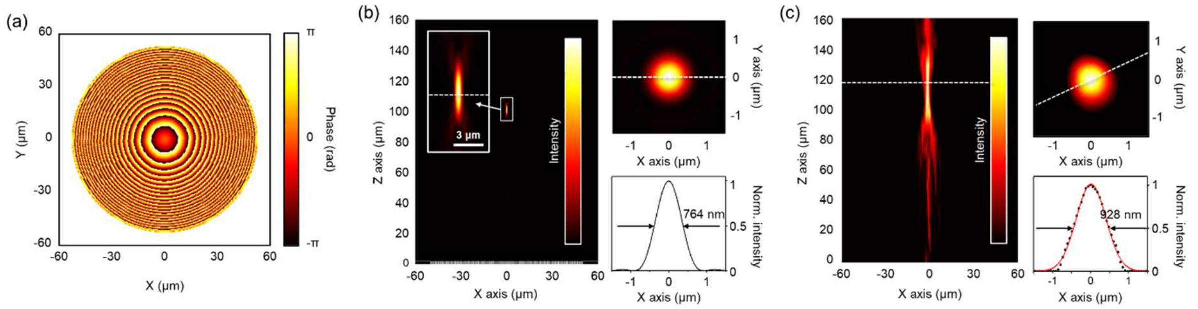

Fig. S7. Single-layer metalens on fiber in 635 nm wavelength (a) Phase map of PMMA metalens which has a target focal length of 100 μm in 635 nm wavelength. The metasurface height is 1800 nm and the lattice of each unit cell is 540 nm. Cause of the metasurface height is under 2000 nm and it can be constructed using a single layer on the fiber apex. (b) Simulation result of metalens calculated with the same method of figure S5(c). The calculated focal length is 98.5 μm, and the calculated FWHM is 764 nm. (c) Experiment result of fabricated metalens on fiber. The fabricated metalens is represented in Figure 2(c). The experimental setup is similar to figure 3(d) that is changed to a 635-nm single-mode fiber (SM600, Thorlabs Inc.) with a core diameter of 4 μm, a visible laser (PL202, Thorlabs Inc.), and visible CMOS camera (HK6.3E3S, K-OPTIC) for measurement of 635 nm wavelength. The FWHM is measured at 928 nm, and the actual focal length is measured to be approximately 121 μm. This is because the light is incident to the metasurface with the divergence angle of SMF, when the NA of SMF used in the experiment is applied to the simulation result and recalculated, the actual focal length in FDTD simulation is calculated to be approximately 129.3 μm, which is closer to the experimental results.

Supplementary Note 8: Single-layer OAM metasurface on fiber in 635 nm wavelength

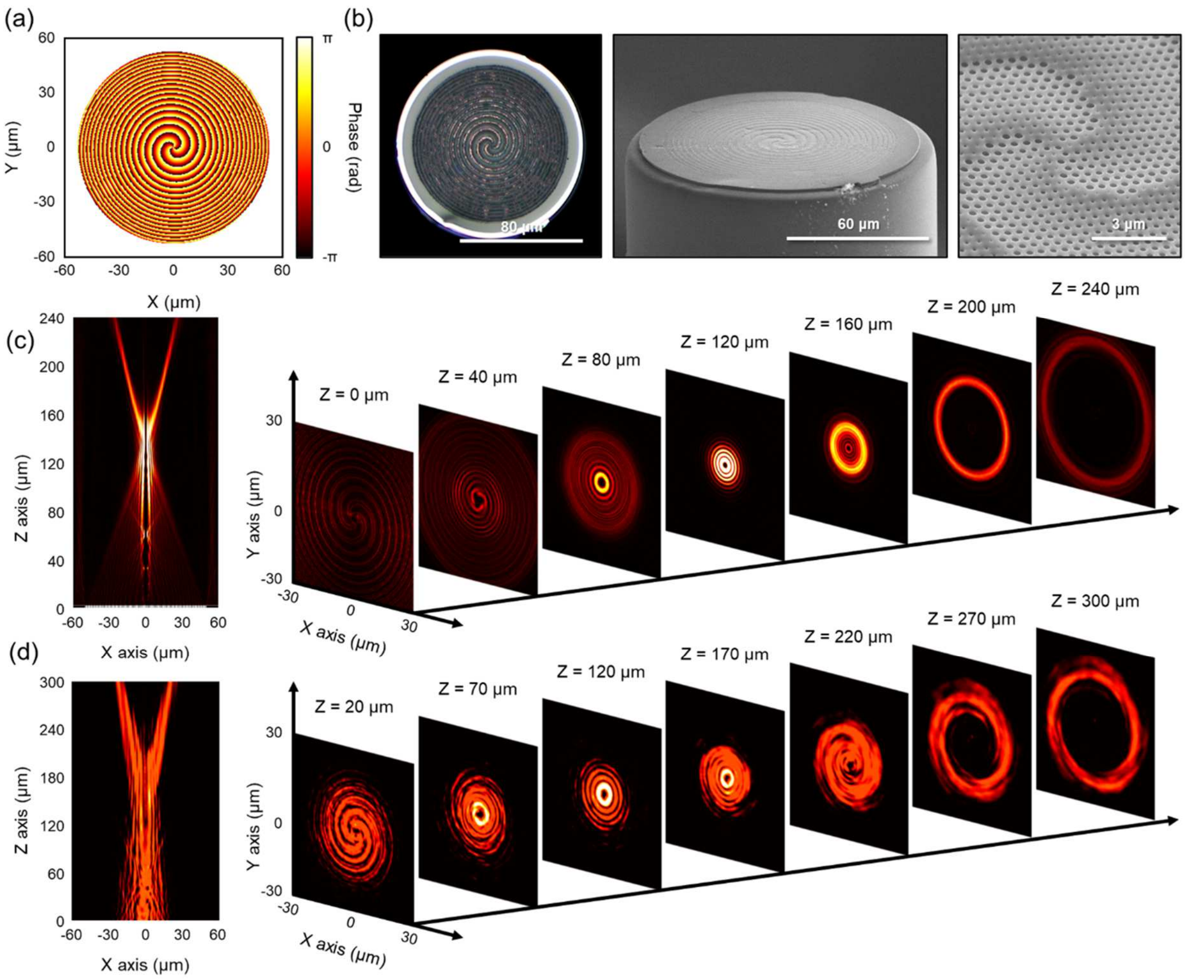

Fig. S8. Single-layer OAM metasurface on fiber in 635 nm wavelength (a) The phase map of OAM metasurface which has a diameter of 105 μm and target wavelength of 635 nm that is fabricated with a target spiral phase plate topological charge value of 3, axicon period 8 μm, and lens focal length 200 μm. (b) The fabrication result of the OAM metasurface transferred to the fiber apex. (c–d) The simulation and experiment results of OAM metasurface.
